# A carrier-free supramolecular nano-twin-drug for overcoming irinotecan-resistance and enhancing efficacy against colorectal cancer

**DOI:** 10.1186/s12951-023-02157-x

**Published:** 2023-10-28

**Authors:** Miaomiao Yuan, Tong Chen, Lu Jin, Peng Zhang, Luoyijun Xie, Shuyi Zhou, Lianfeng Fan, Li Wang, Cai Zhang, Ning Tang, LiHao Guo, Chengmei Xie, Yanhong Duo, Ling Li, Leilei Shi

**Affiliations:** 1 Precision Research Center for Refractory Diseases in Shanghai General Hospital, Shanghai Jiao Tong University School of Medicine, Shanghai, 200025 China; 2 Department of Pharmacy, The Third Affiliated Hospital (The Affiliated Luohu Hospital) of Shenzhen University, 47 Youyi Road, Shenzhen, 518001 China; 3 Department of pharmacology, the Eighth Affiliated Hospital, Joint Laboratory of Guangdong-Hong Kong, Sun Yat-sen University, Universities for Nutritional Metabolism and Precise Prevention and Control of Major Chronic Diseases, Shenzhen, China; 4 School of Pharmaceutical Sciences, Sun Yat-sen University, 510006 Guangzhou, China; 5 Department of Microbiology, Tumor and Cell Biology (MTC), Karolinska Institutet, Stockholm, Sweden

**Keywords:** Carrier-free, Supramolecular self-assembly, Nano-twin-drug, Irinotecan-resistance, Colorectal cancer

## Abstract

Irinotecan (Ir) is commonly employed as a first-line chemotherapeutic treatment for colorectal cancer (CRC). However, tremendous impediments remain to be addressed to surmount drug resistance and ameliorate adverse events. Poly-ADP-Ribose Polymerase (PARP) participates in the maintenance of genome stability and the repair of DNA damage, thus playing a critical role in chemotherapy resistance. In this work, we introduce a novel curative strategy that utilizes nanoparticles (NPs) prepared by dynamic supramolecular co-assembly of Ir and a PARP inhibitor (PARPi) niraparib (Nir) through π-π stacking and hydrogen bond interactions. The Ir and Nir self-assembled Nano-Twin-Drug of (Nir-Ir NPs) could enhance the therapeutic effect on CRC by synergistically inhibiting the DNA damage repair pathway and activating the tumor cell apoptosis process without obvious toxicity. In addition, the Nir-Ir NPs could effectively reverse irinotecan-resistance by inhibiting the expression of multiple resistance protein-1 (MRP-1). Overall, our study underscores the distinctive advantages and potential of Nir-Ir NPs as a complementary strategy to chemotherapy by simultaneously overcoming the Ir resistance and improving the anti-tumor efficacy against CRC.

**Supplementary Information:**

The online version contains supplementary material available at 10.1186/s12951-023-02157-x.

## Introduction

Colorectal cancer (CRC) ranks as the third most frequently diagnosed malignancy and the second leading cause of cancer-related mortality worldwide, with approximately 1.9 million new cases and 920,000 deaths in 2020 [1]. Currently, radical surgical resection is the standard treatment for CRC, although many patients will develop metastatic CRC, which is associated with only 5% relative survival rate [2]. For these metastatic CRC patients, chemotherapy remains an effective treatment option, mainly including 5-fluorouracil, leucovorin, and either irinotecan or oxaliplatin [3]. Irinotecan (Ir), a water-soluble derivative of camptothecin, is FDA-approved as a first-line chemotherapeutic agent for metastatic CRC [4, 5]. Ir functions by inhibiting topoisomerase I, which then affects DNA replication and transcription, leading to cell death [6, 7]. Currently, numerous derivatives of irinotecan are being developed to improve anticancer efficacy and reduce side effects. In the past 25 years, irinotecan is still a must-have drug for the treatment of CRC [8]. However, adverse events always occur in irinotecan therapy because it could be easily effluxed by multidrug resistance (MDR)-associated proteins (MRPs) e.g., in CRC cells [9]. To date, the mechanism of irinotecan resistance remains unclear and requires further investigation. Thus, there is a pressing need to develop innovative approaches to suppress Ir resistance and the associated adverse events.

Irinotecan (Ir) can promote cell death by inducing direct or indirect DNA damage [10], which then can rapidly trigger DNA damage response (DDR) [11]. DDR initiates different repair pathways according to different types of DNA damage, including mismatch repair (MMR), nucleotide excision repair (NER), homologous recombination repair (HRR), base excision repair (BER), and non-homologous end joining (NHEJ) [12, 13]. Downregulation of DDR pathways has emerged as a promising approach since it can augment tumor susceptibility to specific therapeutics and surmount drug resistance, thereby potentiating conventional treatment efficacy [10, 14, 15]. Among others, the poly (ADP-ribose) polymerase (PARP) inhibitors (PARPi) are the best-studied class of DDR inhibitors. Several PARPi have already been approved by the FDA for clinical application, including niraparib (Nir), rucaparib, talazoparib, and Olaparib [16]. Recently, mounting preclinical reports verified the promising synergistic effects of applying PARPi in combination with irinotecan for the treatment of CRC [17–20]. Notably, the inhibition of PARP can confer additional benefits by mitigating Ir-induced intestinal damage, thus minimizing associated adverse events [21]. Therefore, the combination of PARPi and Ir holds potential as an effective strategy for addressing the issues of Ir resistance and adverse events.

Many promising drugs have failed clinical trials for various reasons, for instance, short half-life and high toxicity *in vivo.* As a potential solution, drug delivery systems (DDS) have been extensively investigated for anticancer drug delivery due to benefits such as prolonging the blood circulation time, enhanced tumor accumulation, and reduced side effects [22–28]. Despite great advances in the design of DDS, multiple bottlenecks remain for most of the carrier-assisting DDS, such as their degradation, metabolism, immunogenicity, activated inflammation, complex synthetic procedures, sophisticated design, and low drug loading capacity [29–34]. Recently, the carrier-free drug self-delivery systems composed only of the active drugs themselves have garnered substantial interests [35–37]. Benefiting from the simple and environmentally-friendly preparation procedures, self-delivery nanomedicine not only possesses unique nanoscale advantages but also circumvents the safety concerns associated with additional materials, thereby significantly facilitating their clinical practices [38, 39]. Nevertheless, self-delivery nanomedicine containing PARPi and Ir for CRC has rarely been reported.

**Scheme. 1:**
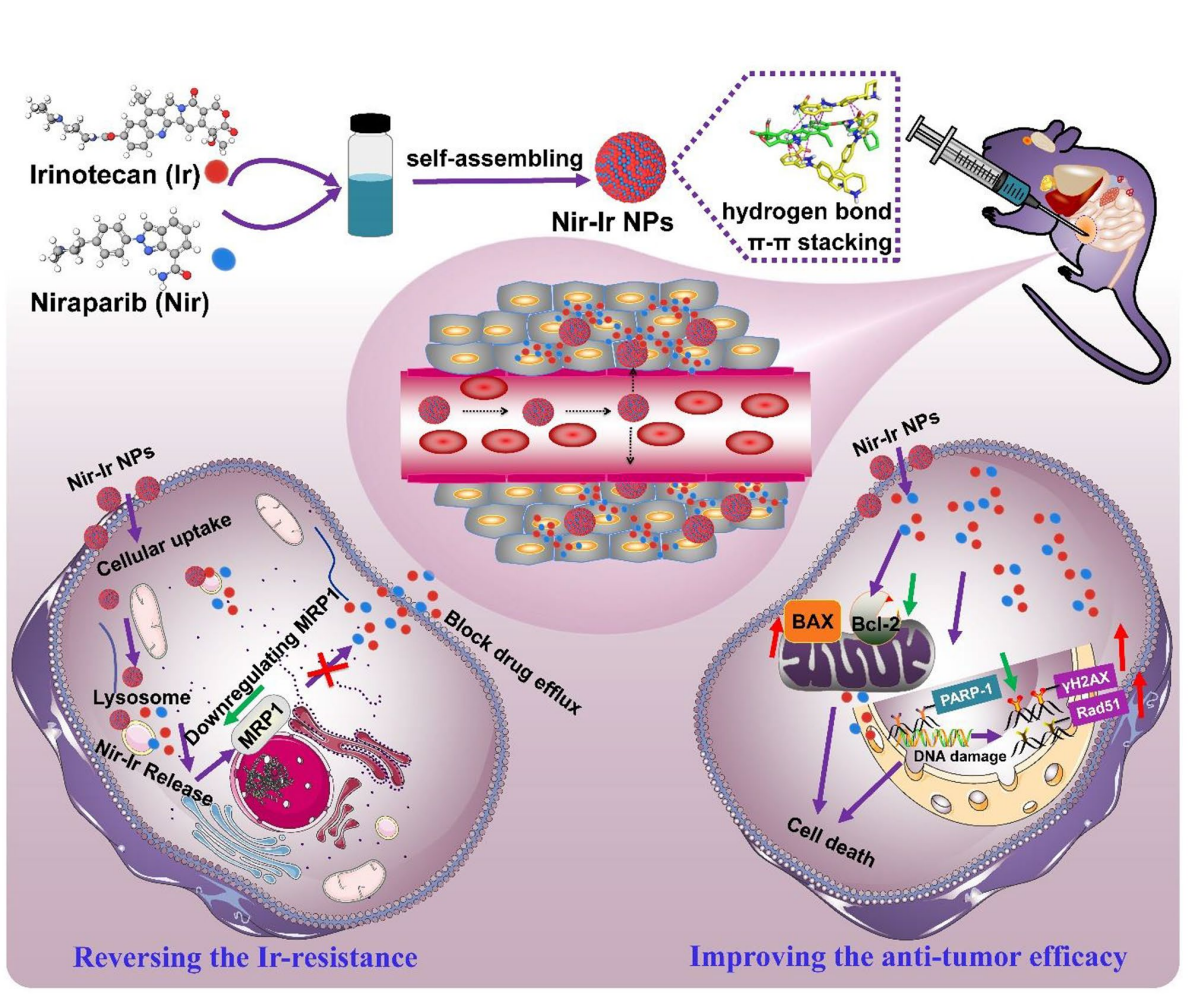
Schematic illustration of self-delivery nanomedicine through π-π stacking and hydrogen bond interactions of Ir and Nir for overcoming Ir-resistance and enhancing efficacy against CRC

Nir is a potent PARPi that has been used in the maintenance therapy of ovarian cancer [40]. In this study, we combined Nir with Ir through a supramolecular assembly process to construct spherical nanoparticles (designated as Nir-Ir NPs). It has been validated that hydrogen bonds, π-π stacking, and hydrophobic interactions participated in their formation and stabilization. Besides, we proved Nir could significantly overcome Ir resistance in cancer cells, eventually improving both the safety and efficacy of CRC chemotherapy (Scheme 1).

## Results and discussion

### Preparation and characterization of Nir-Ir NPs

**Fig. 1:**
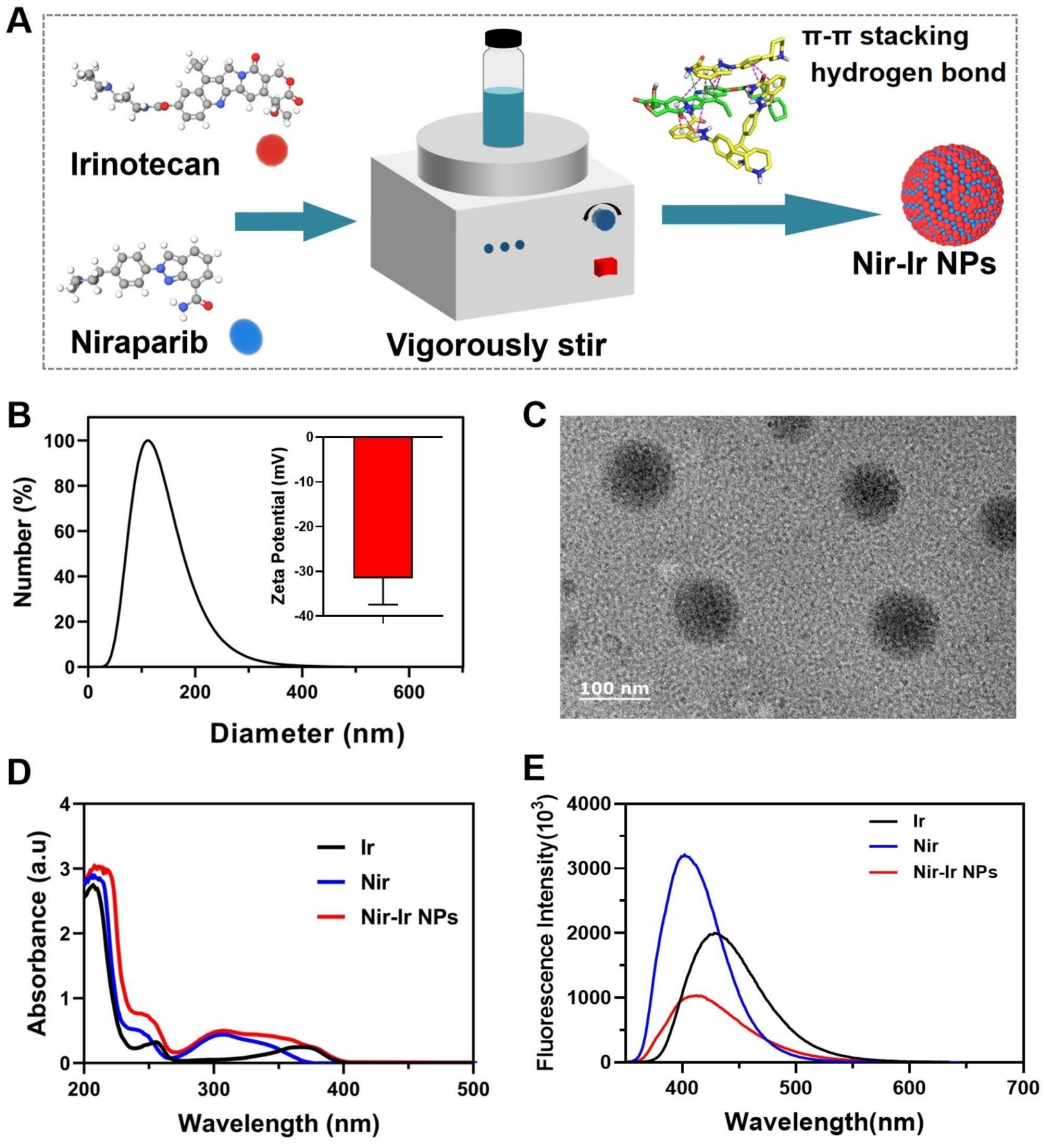
Preparation and characterizations of Nir-Ir NPs. (**A**) Schematic illustration of the preparation of Nir-Ir NPs by the self-assembly of Nir and Ir. (**B**) The hydrodynamic size and zeta potential of Nir-Ir NPs (1 mg/ml in water) determined by DLS analysis. Data were presented as mean values ± SD. (**C**) Representative TEM image of Nir-Ir NPs. (**D**) UV-vis spectra of Ir, Nir and Nir-Ir NPs (1 mg/ml in water). (**E**) Fluorescence emission spectrum of Ir, Nir and Nir-Ir NPs (1 mg/mL in water) with an excitation wavelength of 350 nm

The Nir-Ir NPs were prepared using an amended nanoprecipitation method (Fig. 1A). The supramolecular co-assembly process between Nir and Ir is obviously influenced by their feed ratios (Fig. S1A). Dynamic light scattering (DLS) analysis results show that nanoparticle formation only occurred when the feed ratio of Nir/Ir reached 2:1, while a rise in the hydrodynamic size of the particles from about 100 nm to about 400 nm was observed with increased Nir/Ir feed ratio. Considering that nanoparticles of proper size could preferentially accumulate in tumor through the enhanced permeability and retention (EPR) effect [41], the Nir-Ir NPs prepared with a feed ratio of 2:1 demonstrated a desirable size distribution and thus were chosen for further studies. As shown in Fig. 1B, the hydrodynamic size and zeta potential of Nir-Ir NPs were measured to be about 104 ± 26 nm and − 31 ± 6 mV. Similar results were also observed by transmission electron microscopy (TEM), which showed that the Nir-Ir NPs were uniform spherical nanoparticles with a diameter of about 90 ± 10 nm (Fig. 1C), slightly smaller than the size detected by DLS, possibly because of the swelling of nanoparticles in the hydrated state. These results indicated that Nir-Ir NPs of this size are suitable for further biological application [42].

The stability of Nir-Ir NPs under various conditions was determined by DLS. There were no obvious changes in the particle sizes under a broad concentration range of 0.05-1.0 mg/mL, indicating good stability (Fig. S1B). Furthermore, there was no significant difference in the particle sizes for Nir-Ir NPs in H_2_O, normal saline or DMEM containing 10% FBS (Fig. S1C), and the size of Nir-Ir NPs showed no obvious variations in H_2_O for 7 days (Fig. S1D). Next, the stability of Nir-Ir NPs is further tested in low pH, and high redox condition, simulating the micro-environment of the cancer niche (Fig. S1E,F). Surprisingly, Nir-Ir NPs disassembled into small particles under these conditions, which might contribute to the specific killing effect in tumor region. To sum up, these results evidenced the low critical assembly concentration, good stability and dispersibility of Nir-Ir NPs, which would be beneficial to prolong blood circulation and improve the therapeutic effect.

Subsequently, the ultraviolet-visible (UV-Vis) spectroscopy and fluorescence emission spectrum of Nir-Ir NPs were characterized. As shown in Fig. 1D, the characteristic absorption peaks of Ir were identified at 255 nm and Nir at 306 nm, which were also observed in the synthesized Nir-Ir NPs. Meanwhile, the fluorescence emission spectrum of Nir-Ir shifted compared to that of Ir and Nir (Fig. 1E). Besides, Fourier transform infrared spectroscopy (FTIR) showed the N-H/O-H group peak became sharper in Nir-Ir NPs (Fig. S2A). And the variable temperature FTIR spectroscopy of Nir-Ir NPs revealed that the stretching vibration peak of N-H/O-H bond shifted from 3430 to 3470 nm and became wider when temperature was raised from 25 to 120 °C, indicating that H-bond participated in the formation of Nir-Ir NPs (Fig. S2B).

### The self-assembly mechanisms between Nir and ir using molecular simulations

**Fig. 2:**
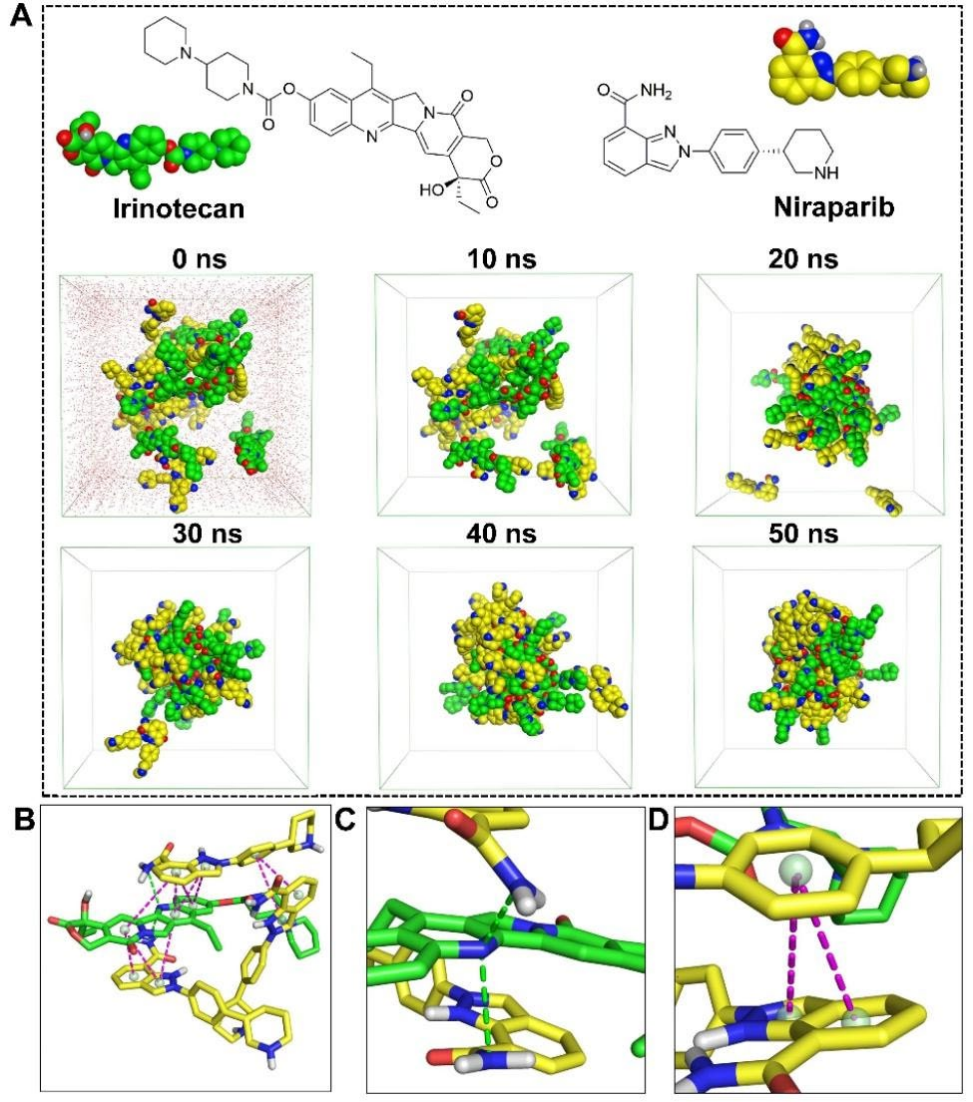
Molecular simulations reveal the self-assembly mechanisms between Ir and Nir. (**A**) Molecular dynamics simulation of Ir and Nir over a 50 ns time scale. (**B**) Stacking manner of Ir and Nir in the assembled state. (**C**) Hydrogen bond interactions between Ir and Nir. (**D**) π-π stacking between Ir and Nir

To further understand the self-assembly mechanism of Nir-Ir NPs, the dynamics process and the interactions between drug molecules were investigated. 34 molecules (Ir: Nir = 1:2.4) were randomly included in a cubic box with a length of 40 Å and subjected to 50 ns of MD simulations. As shown in Fig. 2A, Ir and Nir self-organized and formed assemblies after 50 ns of simulations. We chose one snapshot of the aggregate to illustrate the stacking manner of the two drugs and the representative interactions are shown in Fig. 2B. Two hydrogen bonds were observed between the amide group of Nir and pyridine ring of Ir (Fig. 2C). The π-π stacking were formed between of benzimidazole and benzene ring of Nir (Fig. 2D). Collectively, these results elucidate how intermolecular interactions stabilize the nanosystem.

### In vitro cytotoxicity of Nir-Ir NPs

**Fig. 3:**
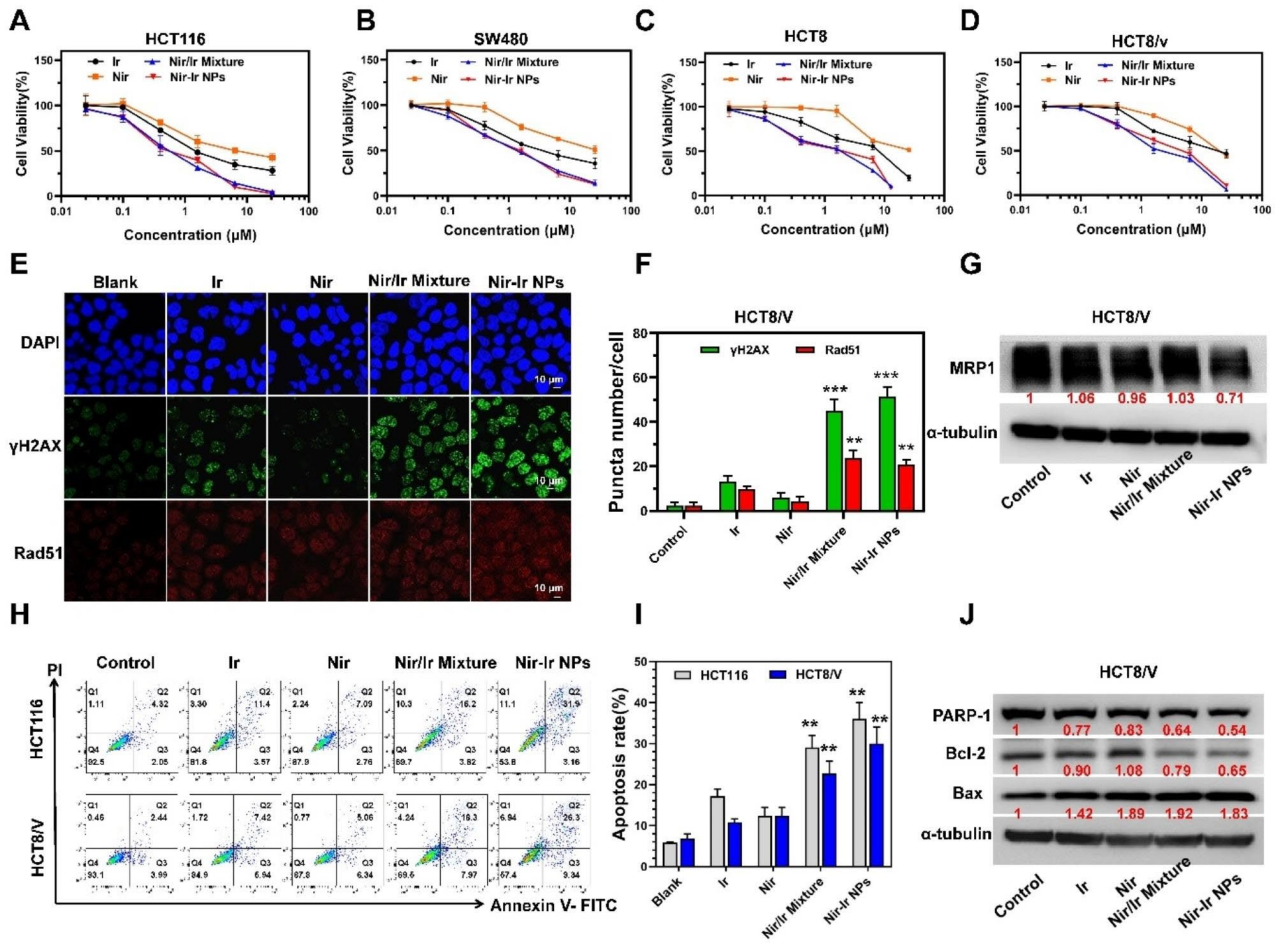
In vitro cytotoxicity of Nir-Ir NPs. **A-D**) Cell viabilities of HCT116 (**A**), SW480 (**B**), HCT8 (**C**) and HCT8/V (**D**) cells after various treatments (control, Ir, Nir Nir/Ir mixture and Nir-Ir NPs) for 72 h. E, F) Immunostaining (**E**) of γH2AX and Rad51 and numbers of each foci (**F**) in HCT8/V cells after various treatments for 24 h. Images were representative of three independent samples. Data were presented as mean values ± SD (n = 3 independent samples). *P < 0.05, **P < 0.01, ***P < 0.001 from two-tailed student’s t test indicated statistical difference compared to the Ir group. **G**) Immunoblotting of the protein expression status of MRP1 in HCT8/V cells after various treatments for 48 h. **H, I**) Representative flow cytometric plot (**H**) and apoptotic percentages (**I**) of tumor cells after various treatments for 48 h using an Annexin V-FITC/PI kit in HCT116 and HCT8/V cells. Data were presented as mean values ± SD (n = 3 independent samples). **P < 0.01 from the two-tailed student’s t test indicated statistical difference compared to the Ir group. **J**) Immunoblot analysis of apoptosis-related proteins expression after various treatments for 48 h (relative expression, fold of α-tubulin )

Cytotoxicity of Nir, Ir, Nir/Ir mixture and Nir-Ir NPs were tested by CCK-8 assay in four different CRC cell lines (HCT116, SW480, HCT8, HCT8/V). Among them, HCT8/V (the vincristine-resistant HCT8 cell line) is an Ir-resistant cell line. From the experimental results, the cell viability decreased gradually with increasing Nir, Ir, Nir/Ir mixture and Nir-Ir NPs dose in four different CRC cell lines and a normal cell line (Fig. 3A-D, Fig. S3A). In Ir/Nir Mixture and Nir-Ir NPs groups, the 50% cellular growth inhibition (IC50) for all four different CRC cell lines was significantly smaller than the Nir and Ir groups, indicating that the combination of Nir and Ir can significantly promote cell death on CRC cell lines (Table S1). A Ir-resistant cell line HCT8/v was constructed to further verify the function of Nir-Ir NPs, and the fold resistance compared with WT cell line is 3.48. Remarkably, the Ir IC50 dosage required for Ir-resistant cell line HCT8/V was 21.35 µg/mL, while the Ir/Nir Mixture and Nir-Ir NPs were only 4.07 and 4.15, respectively, which underscores the potential for addressing the issue of Ir resistance (Table S1). It is noting that Nir-Ir NPs showed quite limited influence on LO2 cells, which is a normal cell line of human liver, indicating potential killing specificity in cancer cells (Fig. S3A). Besides, colony assay also demonstrated that Nir and Ir combination significantly hindered colony growth at low drug doses (Fig. S3B, C). Collectively, these results indicate that Nir-Ir NPs demonstrate a remarkable ability to increase cytotoxicity to CRC cell lines, especially for Ir-resistant cell line.

Both PARPi and Ir could induce persistence of single-strand breaks (SSBs) that can evolve into double-strand breaks (DSBs), and ultimately cause cell death [17, 43]. Therefore, to explore the mechanisms of the Nir-Ir NPs cytotoxicity mentioned above, we investigated whether the enhanced cytotoxicity was related to increased DNA DSBs generation. The γH2AX and Rad51 are DNA DSBs protein biomarkers [44]. As expected, the amount of γH2AX and Rad51 generated by Nir-Ir NPs or Nir/Ir mixture was significantly increased as compared to each free drug groups for the Ir-resistant HCT8/V cell line (Fig. 3E-F) and HCT116 cell line (Fig. S4). To further investigate the effect of reversing Ir-resistance, the multiple resistance protein 1 (MRP1) expression was evaluated in vitro by western blotting. As shown in Fig. 3G, the Nir-Ir NPs significantly reduced the expression of MRP1, suggesting that Nir-Ir NPs can address the issue of Ir-resistance.

Cell apoptosis was further detected by Annexin V-FITC/PI staining in the HCT116 and HCT8/V cell lines. Low-dose Nir/Ir mixture or Nir-Ir NPs induced a higher proportion of apoptotic cells compared to Ir (Fig. 3H-I). Besides, to explore the detailed mechanism of apoptosis, the expression of Bax (a pro-apoptotic protein), Bcl-2 (an antiapoptotic protein) and PAPR-1 (a cleavage substrate of caspase) was evaluated in vitro by western blotting. As shown in Fig. 3J, Nir and Ir combination treatment significantly upregulated the expression of Bax, and suppressed the expression of Bcl-2 and PAPR-1, indicating potentiated apoptotic signaling pathways. Taken together, such evidence shows that Nir-Ir NPs can markedly enhance cytotoxicity of Ir in combination with Nir in different CRC cells and can overcome the Ir-resistance.

### In vitro and In vivo biodistribution

**Fig. 4:**
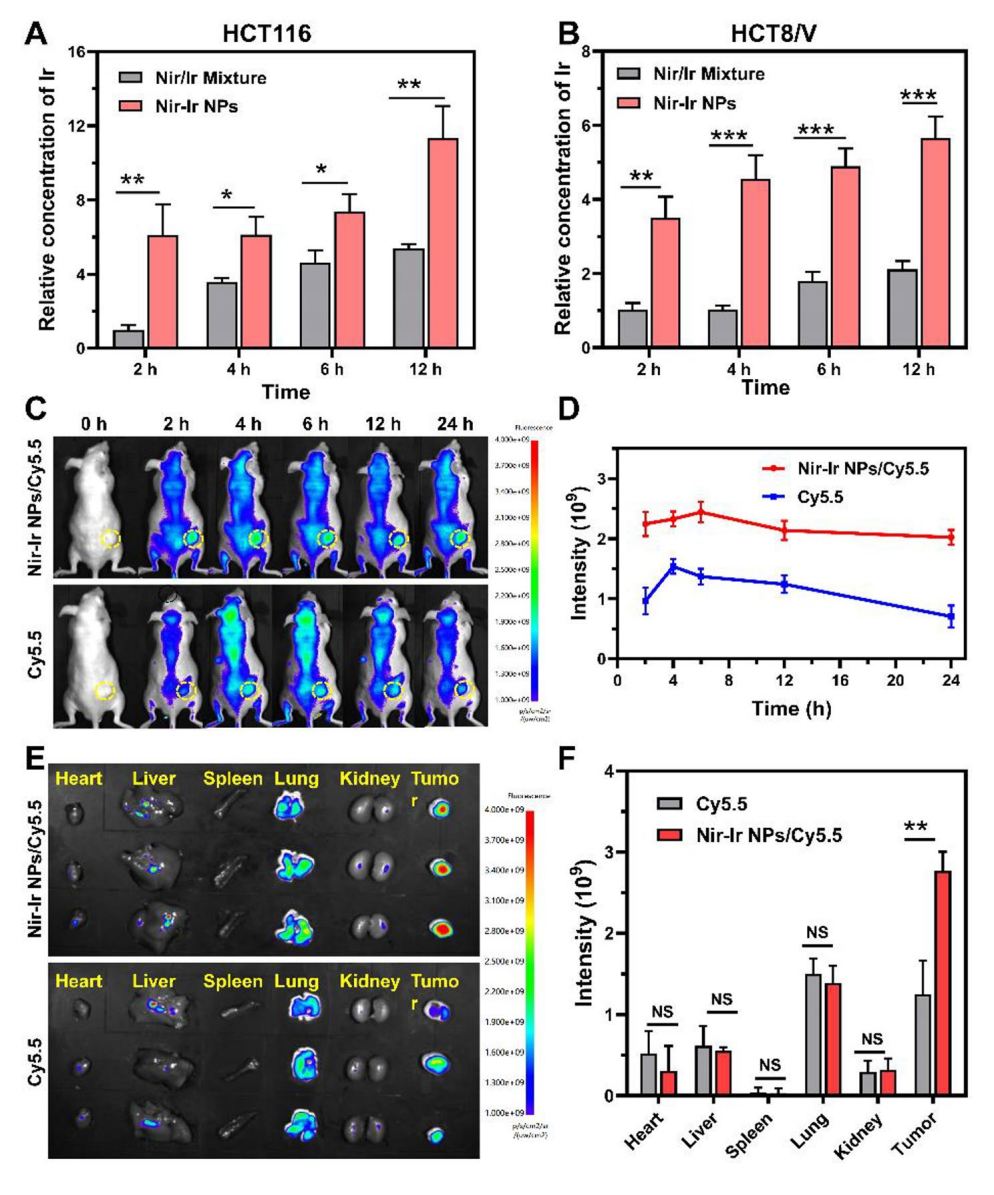
In vitro and In vivo biodistribution. (**A**) Intracellular concentrations of Ir in HCT116 cells measured by flow cytometry at different time points after treated with Nir/Ir mixture and Nir-Ir NPs. Data were presented as mean values ± SD (n = 3 independent samples). *P < 0.05, **P < 0.01, indicated statistical difference between groups from two-tailed student’s t test. (**B**) Intracellular concentration of irinotecan in HCT8/V cells measured by flow cytometry at different time points after treated with Nir-Ir NPs. Data were presented as mean values ± SD (n = 3 independent samples). *P < 0.05, **P < 0.01, ***P < 0.001 indicated statistical difference between groups from two-tailed student’s t test. (**C**) Representative in vivo fluorescence images of mice bearing subcutaneous HCT8/V tumors intravenously injected with Nir-Ir NPs/Cy5.5 or free Cy5.5. Whole-body imaging was performed at predetermined time points. Yellow circles indicate the tumor sites. (**D**) Average fluorescence intensity in the tumor sites at different time points. Data were presented as mean values ± SD (n = 3 mice per group). (**E**) Fluorescence images of ex vivo organs and tumors excised at 24 h. (**F**) Average fluorescence intensity of the organs and tumors. Data were presented as mean values ± SD (n = 3 mice per group). **P < 0.01 from two-tailed student’s t test

Cellular uptake and biological distribution of Nir-Ir NPs were further investigated. As shown in Fig. 4A and B, the cellular uptake of Nir-Ir NPs steadily increased over time and was more efficient than free drugs in vitro for HCT116 and HCT8/V cell lines, which could be ascribed to the gradual internalization of the Nir-Ir NPs by cell endocytosis. Meanwhile, the uptake of free Ir was a passive diffusion dominated process. To assess tumor-specific targeting effects of Nir-Ir NPs, BALB/c nude mice bearing HCT8/V tumors were injected with Nir-Ir NPs/Cy5.5 intravenously using Cy5.5 as a fluorescent tracer (Fig. 4C-F). A strong fluorescence signal was observed at the tumor site of Nir-Ir NPs/Cy5.5-treated mice at 2 h after the injection. Meanwhile, mice injected with free Cy5.5 exhibited much weaker fluorescence at the tumor site, but showed stronger fluorescence signal throughout the whole body. These results indicated the selective accumulation of the nanoparticles in the tumor tissue (Fig. 4C and D). Besides, the Nir-Ir NPs maintained strong fluorescence at the tumor site for 24 h, indicating the retention of the Nir-Ir NPs in tumor sites. On the contrary, fluorescence was hardly observed in mice treated with free Cy5.5 at 24 h post-injection. Quantitative analysis of ex vivo fluorescence images further confirmed that fluorescence intensity of Nir-Ir NPs/Cy5.5 at tumor site is about 2 times higher than that of free Cy5.5 at 24 h post-injection (Fig. 4E and F). Moreover, Nir-Ir NPs/Cy5.5 did not result in obvious accumulation in the major organs including liver, lung, heart, spleen and kidney (Fig. 4E and F). Similar results were also observed in mice bearing subcutaneous HCT116 tumor (Fig. S5). These results together implied that Nir-Ir NPs could dramatically enhance the cellular internalization and intratumoral accumulation, which would substantially promote the effectiveness of antitumor treatment.

### Nir-Ir NPs exhibit excellent antitumor activity in vivo

**Fig. 5:**
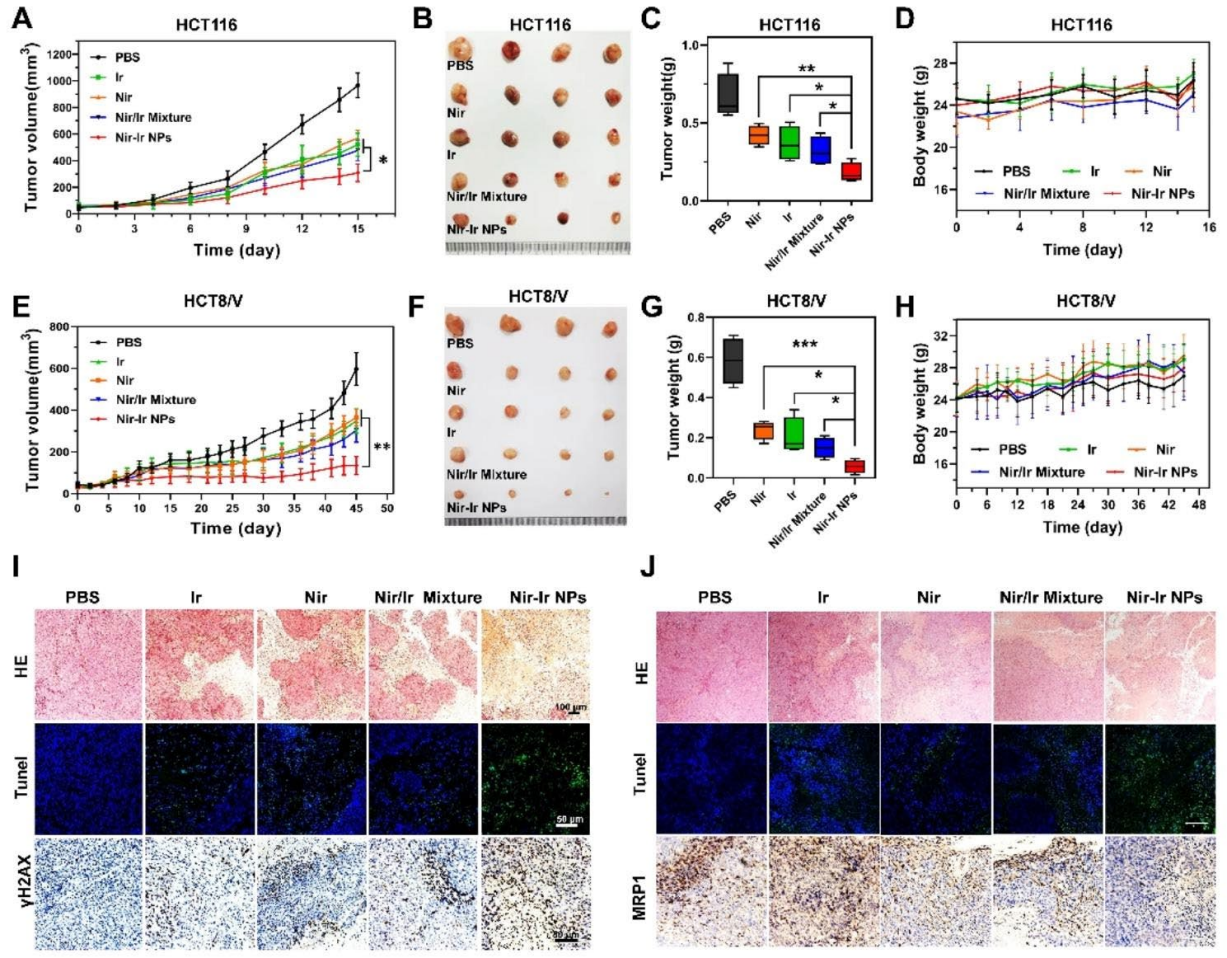
Nir-Ir NPs exhibit excellent antitumor activity in vivo. (**A**) Tumor growth curves of HCT116 subcutaneous tumor-bearing mice following treatment with PBS, Ir, Nir, Nir/Ir mixture or Nir-Ir NPs. Data were presented as mean values ± SD (n = 4 mice per group). (**B**) Representative images of the HCT116 tumors collected at day 15 after treatment for different groups. (**C**) Tumor weight was measured (HCT116 tumor tissue), and the weight of all tumors in each group was compared, *P < 0.05, **P < 0.01 indicated statistical difference between groups from two-tailed student’s t test. (**D**) Body weight record of HCT116 tumor-bearing mice in different groups following treatments. (**E**) Tumor growth curves of Ir-resistant HCT8/V subcutaneous tumor-bearing mice following different treatment. Data were presented as mean values ± SD (n = 4 mice per group). (**F**) Photographs of the HCT8/V tumors collected at day 45. (**G**) Tumor weight was measured (HCT8/V tumor tissue), and the weight of all tumors in each group was compared, *P < 0.05, ***P < 0.001 indicated statistical difference between groups from two-tailed student’s t test. (**H**) Body weight of HCT8/V tumor-bearing mice in different groups following treatments. (**I**) H&E staining images, TUNEL staining and immunohistochemistry assay of γH2AX protein of tumor tissues from the HCT116 tumor-bearing mice. (**J**) H&E staining images, TUNEL staining and immunohistochemistry assay of MRP1 protein of tumor tissues from the HCT8/V tumor-bearing mice. A representative image of four biologically independent animals from each group was shown in (**I, J**). Scale bar: 100 μm

Motivated by the outstanding tumor cell killing capability in vitro and improved tumor-specific targeting in vivo, antitumor activities of Nir-Ir NPs were further evaluated in two mouse xenograft models using HCT116 and HCT8/V, which were Ir-sensitive and Ir-resistant cell lines, respectively. Twenty mice were divided into five groups, and each group was injected every three days with PBS solution, Nir-Ir NPs (1 mg/mL, 200 µL), Nir, Ir, and Nir/Ir mixture of the same drug dosage, respectively. In the HCT116 tumor-bearing mice, the tumor volumes and weight in Nir-Ir NPs group were reduced significantly as compared with the other four groups (Fig. 5A-C). All free drugs (Ir, Nir and Nir/Ir mixture) showed a similar therapeutic effect, highlighting the superiority of Nir-Ir NPs in CRC treatment. During the process of treatment, the body weights of the mice in all groups were not significantly different (Fig. 5D). In consistency with HCT116 tumor-bearing mice, the Nir-Ir NPs group also demonstrated stronger antitumor effects compared with free drugs in the HCT8/V tumor-bearing mice (Fig. 5E-G). The tumor volumes are well controlled throughout the whole therapy in Nir-Ir NPs treatment group, which indicated a reverse of irinotecan-resistance in HCT8/V tumors. The body weights of the HCT8/V tumor-bearing mice in all groups were not significantly different (Fig. 5H).

To further confirm the therapeutic outcome, the histological examination and immunofluorescence staining of HCT116 (Fig. 5I) and HCT8/V (Fig. 5J) tumor tissue were applied. H&E staining of the HCT116 and HCT8/V tumors sections showed that the tumor cells in the Nir-Ir NPs treated group exhibited the most notable cell shrinkage and loss of nuclei, which indicated enhanced cellular apoptosis and death. In contrast, tumors treated with free drugs showed much less intensive apoptosis and necrosis (Fig. 5I-J). Cellular apoptosis was further assessed by deoxyribonucleic acid (DNA) fragmentation via terminal deoxynucleotidyl transferase (TdT)-mediated dUTP nick end labeling (TUNEL) assay, and the apoptosis proportion (green) in the Nir-Ir NPs treated HCT116 and HCT8/V tumors group were significantly higher than that in all other groups (Fig. 5I-J). Immunohistochemical staining of γH2AX demonstrated up-regulated expression in the Nir-Ir NPs treated group comparing to the other groups, indicating the therapeutic effect might result from increased DNA damage (Fig. 5I). Besides, the expression of MRP1 was reduced in Nir-Ir NPs treated group, which benefits the accumulation of Ir in tumor site (Fig. 5J). In summary, Nir-Ir NPs demonstrated excellent anticancer efficacy in vivo and can reverse the Ir-resistance via inhibiting the expression of MRP1.

### In vivo biosafety

**Fig. 6:**
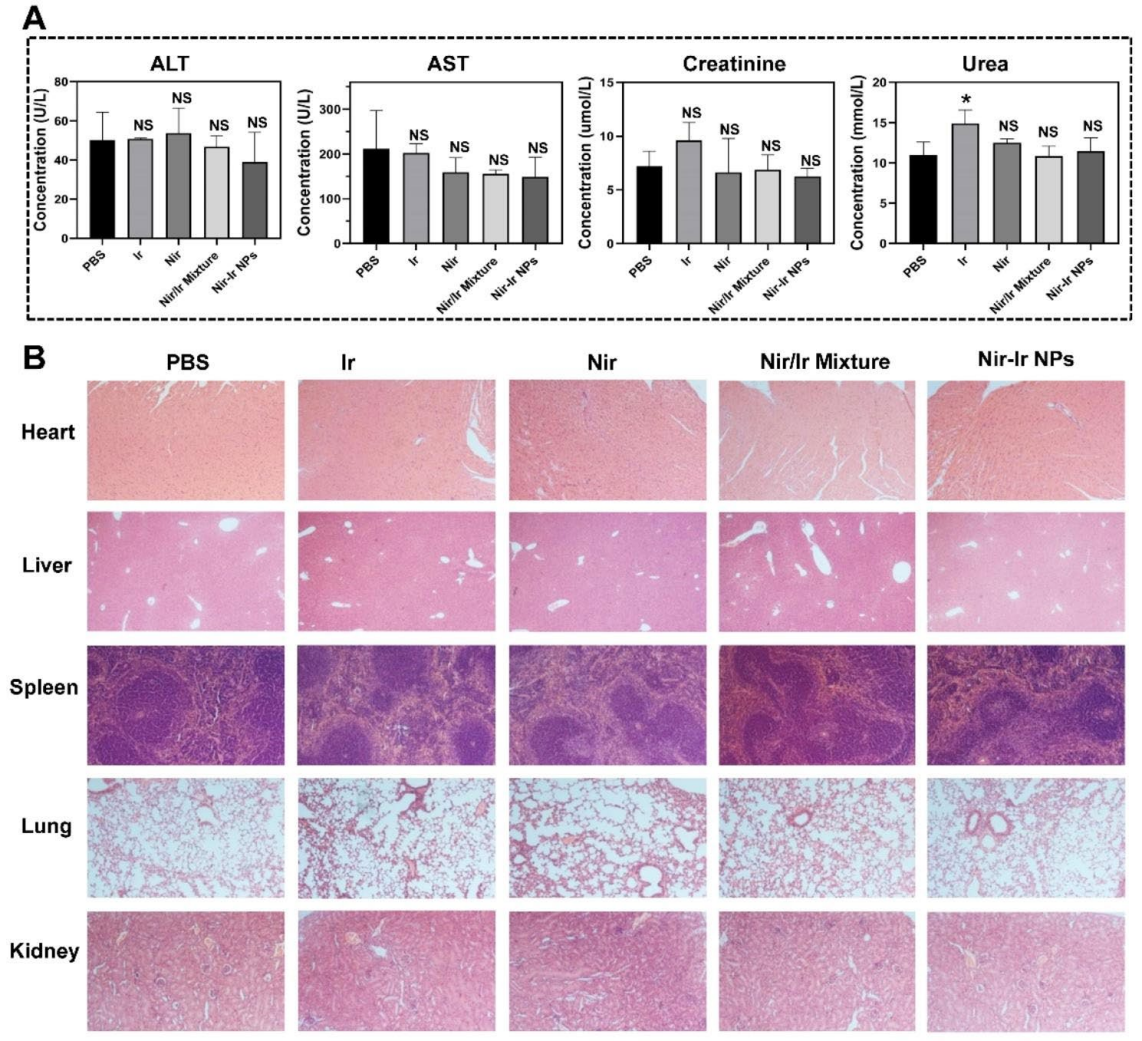
In vivo biosafety. (**A**) Concentrations of ALT, AST, CREA and BUN in serum of HCT8/V subcutaneous tumor-bearing mice receiving different treatments. Data were presented as mean values ± SD (n = 4 independent samples). NS indicates non-significance from two-tailed student’s *t* test compared with the PBS group. (**B**) H&E images of the major organs of HCT8/V cells-bearing mice received different treatment

One of the key challenges for chemotherapy is systemic toxicity. To examine the feasibility of Nir-Ir NPs in clinical application, the toxicity of Nir-Ir NPs was preliminarily assessed by the in-vitro hemo-compatibility assay. And results indicated high compatibility of Nir-Ir NPs with peripheral blood cells (Fig. S6). Moreover, the toxicity of Nir-Ir NPs was further assessed in two mouse xenograft models using Ir-resistant HCT8/V (Fig. 6) and Ir-sensitive HCT116 cell lines (Fig. S7). When compared with the PBS control group, the injection of Nir-Ir NPs in mice induced no notable difference, where the level of alanine transaminase (ALT), aspartate aminotransferase (AST), creatinine (CREA) and blood urea nitrogen (BUN) in serum were maintained within the normal range (Fig. 6A, and Fig. S7A ). Meanwhile, the injection of Nir-Ir NPs in two mouse xenograft models caused no serious damage to heart, liver, lung and kidney (Fig. 6B, and Fig. S7B). Therefore, Nir-Ir NPs can not only improve the tumor-specific delivery of chemodrugs, but also reduce their systemic toxicity.

## Conclusion

Ir resistance and adverse events are major reasons that limit its clinical therapeutic efficiency in metastatic CRC patients. In this study, we successfully developed a novel supramolecular nano-twin-drug through dynamic supramolecular-assembly of Ir and Nir to address the Ir resistance and improve the safety of CRC chemotherapy. The supramolecular nano-twin-drug was stabilized by multiple intermolecular interactions, including hydrogen bond and π-π stacking without introducing external nanocarriers or molecular modification. After being treated by Ir-Nir NPs, drug resistance was reversed by down-regulation of MRP1 in CRC tumor cells. More importantly, the in vivo anti-tumor efficacy was remarkably enhanced in both HCT116 and HCT8/V tumor-bearing mice. Overall, for future chemotherapy in CRC patients, our work presents a practical and effective complementary treatment nanoplatform which can simultaneously overcome Ir resistance and reduce adverse events.

## Methods

### Materials and reagents

Ir and Nir were purchased from Meilun Biotechnology Co. Ltd (Dalian, China). Dimethyl sulfoxide (DMSO) and annexin V-FITC/PI apoptosis detection kit were purchased from Sigma-Aldrich (Shanghai, China). Cyanine5.5 (Cy5.5) were purchased from MedChemExpress (NJ, USA). Dulbecco’s modified Eagle’s medium (DMEM), Roswell Park Memorial Institute (RPMI) 1640 medium, fetal bovine serum (FBS), PBS, trypsin-EDTA and penicillin/streptomycin were purchased from Thermo Fisher Scientific (Waltham, USA). The CCK-8 kit was purchased from Beyotime Biotechnology (Shanghai, China). All the chemicals were used as supplied without further purification.

### Cells and animals

Human HCT116, SW480, HCT8, HCT8/V authenticated colorectal cancer cell lines, and LO2 a normal cell line of human liver cells were obtained from the Cell Bank of the Chinese Academy of Sciences (Shanghai, China), with no mycoplasma contamination. All cells were cultured in recommended medium with 10% FBS at 37 ◦C in an incubator with 5% CO_2_. 5-week-old female BALB/c nude mice (18–22 g, SPF grade) were purchased from GemPharmatech (Nanjing, China). The animal protocols were approved by the Ethics Review Committee for Animal Experimentation for The Eighth Affiliated Hospital, Sun Yat-sen University (Shenzhen, China) (Approved number: 2022-009-01).

### Preparation and characterization of the Nir-Ir supramolecular nanoparticles

Nir-Ir NPs were obtained via an amended nanoprecipitation method. Briefly, Ir and Nir were dissolved in dimethyl sulfoxide (DMSO) with appropriate ratio, and the mixture was added to deionized water (c = 1 mg/mL). The resultant solution was stirred slightly at room temperature for 30 min and stored at 4 °C for further use. The morphology of the resultant nanoparticles was studied using a transmission electron microscopy (TEM, JEOL JEM-2100 F, Japan). The hydrodynamic diameter and zeta potential of Nir-Ir NPs were measured by dynamic light scattering (DLS) using a NanoBrook 90Plus PALS. UV-Vis spectrophotometer was used to obtain the absorption spectra, and a Thermo Scientific Varioskan LUX was used to obtain the fluorescence emission spectra. The fourier transform infrared spectroscopy (FTIR) spectrum was scanned by the spectrometer (Nicolet 6700, thermo scientific, USA). Besides, to track Nir-Ir NPs, Cy5.5 was added to DMSO solution of irinotecan and niraparib (1% in total mole), and the other steps were the same as described above.

### Stability test

To test colloidal stability, freshly prepared Nir-Ir NPs solution with a concentration of 1 mg/mL was stored at room temperature (RT) for 7 days, and the size change of NPs was recorded by DLS as described above. The colloidal stability of Nir-Ir NPs was also evaluated under incubation with normal saline and DMEM containing 10% FBS for 24 h. For low pH stability test, the solution of Nir-Ir NPs (1 mg/mL) was adjusted to pH 6.5. And the high redox condition was simulated by adding 10 µL H_2_O_2_ (30% v/v) into the Nir-Ir NPs solution.

### Molecular dynamics (MD) simulation

The structures of Ir and Nir were optimized under B3LYP/6-31G* by Gaussian09 package. After that, the HF/6-31G* method and basis set were used to calculate the electrostatic potential (ESP) and then the result was employed to calculate the restricted ESP(RESP)2 charge. MMFF94x Force Field parameters were used for characterizing those two drugs. 24 Niraparib and 10 irinotecan molecules were initially packed randomly by PACKMOL in a cubic box with a length of 40 Å. Then the mixture was neutralized by adding sodium/chlorine counter ions and solvated in a cuboid box of TIP3P water molecules with solvent layers 10 Å between the box edges and solute surface. MD simulation was performed using AMBER18. The complex was centered in a box of 10 Å margin solvated by the TIP3P water model. Periodic boundary condition (PBC) was set to allow free motion along the 3D lattice. Nonbonded van der Waals interactions were calculated using the Lennard-Jones 12 − 6 potentials with a 10 Å cutoff, while long-range electrostatics were treated using the Particle Mesh Ewald (PME)algorithm [45]. The SHAKE algorithm was applied to constrain bonds involving hydrogen atoms [46]. To remove improper atom contacts, a steepest descent minimization of 500,000 steps was performed. And then the system was heated up to 300 K in 50 ps. Subsequently, a two-step equilibration phase was carried out to simulate constant volume (NVT) and constant pressure (NPT) ensembles, respectively. The phase was simulated for 100 ps at 300 K using the Langevin dynamics method to control the temperature with collision frequency of 1.0 ps-1. At last, a 50 ns MD simulation was conducted with the integration time step of 2.0 fs.

### In vitro cytotoxicity study

Cytotoxicity was analyzed by the CCK-8 assay according to the manufacturer’s instructions. Briefly, for each cell line, 1–5 × 10^3^ cells per well were seeded in a 96-well plate, and then incubated overnight. The cells were treated with different concentrations of Ir, Nir, Ir/Nir mixture or Nir-Ir NPs. After 72 h incubation, the medium was replaced, and the cell viability was detected using the CCK-8 kit. The absorbance at 450 nm of each well was recorded on a microplate reader. Untreated cells were used as controls. IC50 values were determined by CompuSyn 1.0 software. Colony formation assay was used to analyze the long-term proliferative potential of cell lines following treatments with Ir, Nir, Ir/Nir mixture and Nir-Ir NPs. 4–10 × 10^2^ cells per well were seeded in 6-well plates and incubated with the drugs with the same Nir or Ir concentration (Ir, 0.2 µM and Nir, 0.4 µM) for 72 h. The medium was replaced every 3 days. After 2 weeks, cells were fixed with 4% paraformaldehyde for 20 min, and then stained with 0.1% crystal violet for 30 min.

### Immunofluorescence and annexin-V FITC/PI assay

To study the DNA damage induced by Nir-Ir NPs, 5 × 10^4^ cells/well were seeded on a confocal dish and treated with Ir, Nir, Ir/Nir mixture or Nir-Ir NPs with the same Nir or Ir concentration (Ir, 0.2 µM and Nir, 0.4 µM) for 24 h. Cells were washed in PBS, fixed with 4% paraformaldehyde (PFA) and permeabilized with 0.2% Triton X-100/PBS solution for 10 min. Blocking was performed using 1% BSA for 30 min at room temperature. Cells were incubated with rabbit primary anti-phospho-Histone-H2AX antibody (Cell Signaling Technology Cat# 9718) and mouse anti-RAD51 antibody (Genetex Cat# GTX70230) in PBS overnight at 4 °C. Secondary goat anti-rabbit Alexa Fluor 488-conjugated (Thermo Fisher Scientific Cat# A-11,008) and goat anti-mouse Alexa Fluor 555-conjugated (Thermo Fisher Scientific Cat# A-21,424) antibodies were added for 1 h at RT after PBS wash once. Cells were then incubated with DAPI (Thermo Fisher Scientific, Cat# D1306) in PBS for 10 min in the dark. Images were collected under a Zeiss LSM 800 laser confocal scanning microscope. To analyze the cellular apoptosis induced by Nir-Ir NPs, 1*10^5^ cells/well cells were plated in 6-well plates and cultured overnight. Then cells were incubated with the drugs as described above for 48 h. Afterwards, cells were washed with PBS and stained by annexin-V FITC and propidium iodide (PI) according to the manufacturer’s protocol. The fluorescence intensity of cells was measured by a BD LSRFortessa flow cytometry in green channel for annexin V-FITC and red channel for PI, respectively.

### Western blotting and quantitative PCR

3 × 10^5^ cells/well cells were seeded in 6-well plates and cultured overnight. Cells were treated with Ir, Nir, Nir/Ir mixture or Nir-Ir NPs for 48 h. Then the cells were washed with PBS and lysed by RIPA buffer containing protease/phosphatase inhibitor cocktails (Beyotime Cat# P1045). Cell lysates were centrifuged, and the supernatants were loaded on SDS-PAGE, followed by transferring to the PVDF membrane (BIORAD, Cat# 1704156). The blots ware blocked with TBST containing 5% bovine serum albumin (BSA) for 1 h and incubated with primary antibodies against γH2Ax (Cell Signaling Technology Cat# 9718), Bax (Abcam Cat# ab182733), Bcl-2 (Cell Signaling Technology Cat# 3498), PARP-1 (Cell Signaling Technology Cat# 9532) and MRP-1 (Abcam Cat# ab233383) at 4 ◦C overnight. Then, membranes were washed with TBST and incubated with the HRP-linked antibody at RT for 1 h. A ChemiDoc Imager system (Bio-Rad, ChemiDoc Touch) was used to detect the bands of specific proteins. Total RNA was isolated from SW480 cells with Trizol (Invitrogen, USA). Reverse transcription was performed with a PrimeScript reverse transcription reagent kit (Takara, Japan). After cDNA was amplified in Thermal Cycler (Bio-Rad, C1000 Touch), quantitative PCR was performed with TB Green Premix Ex Taq (Takara, Japan) and a fluorescence quantitative real-time PCR machine (Roche, LightCyele480). GAPDH mRNA was used as a reference. Primers were: hCCL5: 5’ - CCTGCTGCTTTGCCTACATTGC-3’ (sense) and 5’ - ACACACTTGGCGGTTCTTTCGG-3’ (antisense); hCXCL10: 5’GTGGCATTCAAGGAGTACCTC-3’ (sense) and 5’ - TGATGGCCTTCGATTCTGGATT-3’(antisense); hIFNB1: 5’-CTGCATTACCTGAAGGCCAAG-3’ (sense) and 5’- TTGAAGCAATTGTCCAGTCCC-3’ (antisense); hGAPDH: 5’- GCACCGTCAAGGCTGAGAAC-3’ (sense) and 5’-TGGTGAAGACGCCAGTGGA-3’(antisense).

### Cellular uptake and in vivo biodistribution of Nir-Ir NPs

To estimate the endocytosis of Nir-Ir NPs, cells were seeded in a 6-well plate at a density of 3 × 10^5^ cells/well and incubated overnight. Then the cells were treated with Nir/Ir mixture or Nir-Ir NPs for another 2 to 12 h. After that, the fluorescence of Ir or Nir-Ir NPs in the cells were analyzed by flow cytometry using a specific channel (405 nm laser, 450 nm/40 nm filter). Fluorescence imaging were performed to study the in vivo biodistribution of Nir-Ir NPs. In brief, tumor-bearing mice were subcutaneously injected with 5 × 10^6^ HCT8/V or HCT116 cells into the right flank of female BALB/c nude mice. When the tumor volume exceeded 100 mm^3^, Cy5.5-labelled Nir-Ir NPs or free Cy5.5, with an equivalent Cy5.5 dose of 0.2 mg/kg, were intravenously injected into the tumor-bearing mice (n = 3). Fluorescence signals were detected at 2 h, 4 h, 6 h, 12 h, 24 h post-intravenous injection by an in vivo fluorescence imaging system (Biolight Biotechnology, AniView100) with excitation at 630 nm and emission at 680 nm. Then the mice were sacrificed at 24 h post-injection to collect the tumors and major organs. The average fluorescence intensities from Cy5.5 in tumors and major organs were evaluated to reveal the in vivo biodistribution.

### Hemocompatibility evaluation

The whole blood sample was collected from a BALB/c mice into an EDTA anti-coagulated tube, and then was supplemented with 1 mL PBS to wash once at 2000 rpm for 10 min. After removing the supernatant, 10 mL PBS was added to dilute the blood sample. Then 200 µL of the diluted blood cells were co-incubated with 1 mL PBS (negative control), deionized water (positive control), or various concentrations of Nir-Ir NPs diluted in PBS (3.8, 7.7, 12.5, 25, 50 and 100 µM) for 2 h at 37℃. Afterwards, samples were centrifuged at 12,000 rpm for 10 min, and the supernatant was added into a 96-well plate to detect the absorbance at 570 nm. The calculation method of hemolysis rate is hemolysis ratio (%) = (A (sample570 nm) - A (negative, 570 nm))/(mean value of A (positive, 570 nm)-A (negative, 570 nm)) × 100% .

### In vivo therapeutic efficacy and biosafety

In vivo antitumor efficacy of Nir-Ir NPs was studied in HCT116 and HCT8/V tumor models. A total of 5 × 10^6^ cells were resuspended in 200 µL PBS and implanted subcutaneously into the right flank of 20 mice for each cell line. The mice were randomly divided into five groups when the tumors reached a volume of 75–100 mm^3^, with 4 mice in each group, and were intravenously injected with: (i) PBS; (ii) Nir-Ir NPs (200 uL, 1 mg/mL); (iii) Ir, (iv) Nir, (v) Ir/Nir mixture (equivalent Ir or Nir dose) every three days. The volume of tumors was measured every other day and calculated by the following equation: V = L × W^2^/2. Mice were weighed every three days. When the tumor diameter reached 15 mm, mice were euthanized to collect whole blood, tumors, and major organs (liver, heart, kidney, lung, spleen) for further analysis. The tumors were weighed and photographed. The serum alanine aminotransferase (ALT), aspartate aminotransferase (AST), blood urea nitrogen (BUN) and creatinine (CREA) were measured by serum biochemical analysis to reveal the long-term toxicity to the liver and kidney. The tissues were fixed with 4% paraformaldehyde solution and embedded in paraffin, followed by staining with hematoxylin and eosin (H&E) for further observation by optical microscopy. The tumor sections were also stained by the terminal deoxynucleotidyl transferase dUTP nick end labeling (TUNEL), γH2AX and MRP1 for histology studies.

### Statistical analyses

Data were analyzed using GraphPad Prism 9.0 software. Statistical analysis was performed by Student’s t-test. The data were presented as means ± standard deviation (SD) unless otherwise indicated. Significant differences were considered if P values < 0.05; * for P < 0.05, ** for P < 0.01, *** for P < 0.001 and NS. for non-significant.

### Electronic supplementary material

Below is the link to the electronic supplementary material.

Supplementary Material 1

## References

[1] Sung H, Ferlay J, Siegel RL, Laversanne M, Soerjomataram I, Jemal A, Bray F (2021). Global Cancer statistics 2020: GLOBOCAN estimates of incidence and Mortality Worldwide for 36 cancers in 185 countries. Cancer J Clin.

[2] Mulsow J, Merkel S, Agaimy A, Hohenberger W (2011). Outcomes following Surgery for Colorectal cancer with synchronous peritoneal metastases. J Br Surg.

[3] Kuipers EJ, Grady WM, Lieberman D, Seufferlein T, Sung JJ, Boelens PG, van de Velde CJH, Watanabe T (2015). Colorectal cancer. Nat Reviews Disease Primers.

[4] Du Y, Zhang W, He R, Ismail M, Ling L, Yao C, Fu Z, Li X (2017). Dual 7-ethyl-10-hydroxycamptothecin conjugated phospholipid prodrug assembled liposomes with in vitro anticancer effects. Bioorg Med Chem.

[5] Si J, Zhao X, Gao S, Huang D, Sui M (2019). Advances in delivery of Irinotecan (CPT-11) active metabolite 7-ethyl-10-hydroxycamptothecin. Int J Pharm.

[6] Ebrahimnejad P, Dinarvand R, Sajadi A, Jaafari MR, Nomani AR, Azizi E, Rad-Malekshahi M, Atyabi F (2010). Preparation and in vitro evaluation of actively targetable nanoparticles for SN-38 delivery against HT-29 cell lines. Nanomed Nanotechnol Biol Med.

[7] Xu Y, Villalona-Calero M (2002). Irinotecan: mechanisms of Tumor resistance and novel strategies for modulating its activity. Ann Oncol.

[8] Bailly C (2019). Irinotecan: 25 years of cancer treatment. Pharmacol Res.

[9] Negi LM, Jaggi M, Joshi V, Ronodip K, Talegaonkar S (2015). Hyaluronic acid decorated lipid nanocarrier for MDR modulation and CD-44 targeting in colon adenocarcinoma. Int J Biol Macromol.

[10] Vitiello PP, Martini G, Mele L, Giunta EF, De Falco V, Ciardiello D, Belli V, Cardone C, Matrone N, Poliero L (2021). Vulnerability to low-dose combination of irinotecan and niraparib in ATM-mutated Colorectal cancer. J Experimental Clin Cancer Res.

[11] Mei C, Sun ZE, Tan LM, Gong JP, Li X, Liu ZQ (2022). eIF3a-PPP2R5A-mediated ATM/ATR dephosphorylation is essential for irinotecan-induced DNA damage response. Cell Prolif.

[12] Shi C, Qin K, Lin A, Jiang A, Cheng Q, Liu Z, Zhang J, Luo P (2022). The role of DNA damage repair (DDR) system in response to immune checkpoint inhibitor (ICI) therapy. J Experimental Clin Cancer Res.

[13] Reilly NM, Novara L, Di Nicolantonio F, Bardelli A (2019). Exploiting DNA repair defects in Colorectal cancer. Mol Oncol.

[14] Ferri A, Stagni V, Barilà D (2020). Targeting the DNA damage response to Overcome Cancer Drug Resistance in Glioblastoma. Int J Mol Sci.

[15] Xiao Y, Lin FT, Lin WC (2021). ACTL6A promotes repair of cisplatin-induced DNA damage, a new mechanism of platinum resistance in cancer. Proc Natl Acad Sci USA.

[16] Pilié PG, Tang C, Mills GB, Yap TA (2019). State-of-the-art strategies for targeting the DNA damage response in cancer. Nat Reviews Clin Oncol.

[17] Genther Williams SM, Kuznicki AM, Andrade P, Dolinski BM, Elbi C, O’Hagan RC, Toniatti C (2015). Treatment with the PARP inhibitor, niraparib, sensitizes Colorectal cancer cell lines to irinotecan regardless of MSI/MSS status. Cancer Cell Int.

[18] Davidson D, Wang Y, Aloyz R, Panasci L (2013). The PARP inhibitor ABT-888 synergizes irinotecan treatment of colon Cancer cell lines. Investig New Drugs.

[19] Tahara M, Inoue T, Sato F, Miyakura Y, Horie H, Yasuda Y, Fujii H, Kotake K, Sugano K (2014). The Use of Olaparib (AZD2281) potentiates SN-38 cytotoxicity in Colon Cancer cells by Indirect Inhibition of Rad51-Mediated repair of DNA double-strand BreaksOlaparib Potentiates SN-38 sensitivity in Colon Cancer cells. Mol Cancer Ther.

[20] Augustine T, Maitra R, Zhang J, Nayak J, Goel S (2019). Sensitization of Colorectal cancer to irinotecan therapy by PARP inhibitor rucaparib. Investig New Drugs.

[21] Tentori L, Leonetti C, Scarsella M, Muzi A, Mazzon E, Vergati M, Forini O, Lapidus R, Xu W, Dorio AS (2006). Inhibition of poly (ADP-ribose) polymerase prevents irinotecan‐induced intestinal damage and enhances irinotecan/temozolomide efficacy against colon carcinoma. FASEB J.

[22] Sargazi S, Mukhtar M, Rahdar A, Barani M, Pandey S, Díez-Pascual AM (2021). Active targeted nanoparticles for delivery of poly(ADP-ribose) polymerase (PARP) inhibitors: a preliminary review. Int J Mol Sci.

[23] Passero FC, Grapsa D, Syrigos KN, Saif MW (2016). The safety and efficacy of Onivyde (irinotecan liposome injection) for the treatment of metastatic Pancreatic cancer following gemcitabine-based therapy. Expert Rev Anticancer Ther.

[24] Xue X, Qu H, Li Y (2022). Stimuli-responsive crosslinked nanomedicine for cancer treatment. Exploration.

[25] Sun Z, Hou Y (2023). Intelligent micro/nanorobots for improved Tumor therapy. BMEMat.

[26] Zheng P, Ding J (2022). Calcium ion nanomodulators for mitochondria-targeted multimodal cancer therapy. Asian J Pharm Sci.

[27] Zheng P, Ding B, Shi R, Jiang Z, Xu W, Li G, Ding J, Chen X (2021). A multichannel Ca2 + nanomodulator for Multilevel mitochondrial Destruction-mediated Cancer Therapy. Adv Mater.

[28] Chen J, Jiang Z, Zhang YS, Ding J, Chen X (2021). Smart transformable nanoparticles for enhanced Tumor theranostics. Appl Phys Reviews.

[29] Torchilin VP (2014). Multifunctional, stimuli-sensitive nanoparticulate systems for drug delivery. Nat Rev Drug Discovery.

[30] Fan W, Yung B, Huang P, Chen X (2017). Nanotechnology for multimodal synergistic cancer therapy. Chem Rev.

[31] Dai Y, Xu C, Sun X, Chen X (2017). Nanoparticle design strategies for enhanced anticancer therapy by exploiting the tumour microenvironment. Chem Soc Rev.

[32] Landesman-Milo D, Peer D (2012). Altering the immune response with lipid-based nanoparticles. J Controlled Release.

[33] Dewhirst MW, Secomb TW (2017). Transport of Drugs from blood vessels to tumour tissue. Nat Rev Cancer.

[34] Wang B, He X, Zhang Z, Zhao Y, Feng W (2013). Metabolism of nanomaterials in vivo: blood circulation and organ clearance. Acc Chem Res.

[35] Qin S-Y, Zhang A-Q, Cheng S-X, Rong L, Zhang X-Z (2017). Drug self-delivery systems for cancer therapy. Biomaterials.

[36] Zhao L-P, Zheng R-R, Chen H-Q, Liu L-S, Zhao X-Y, Liu H-H, Qiu X-Z, Yu X-Y, Cheng H, Li S-Y (2020). Self-delivery nanomedicine for O2-economized photodynamic Tumor therapy. Nano Lett.

[37] Zheng R-R, Zhao L-P, Liu L-S, Deng F-A, Chen X-Y, Jiang X-Y, Wang C, Yu X-Y, Cheng H, Li S-Y (2021). Self-delivery nanomedicine to overcome drug resistance for synergistic chemotherapy. Biomaterials Sci.

[38] Kasai H, Murakami T, Ikuta Y, Koseki Y, Baba K, Oikawa H, Nakanishi H, Okada M, Shoji M, Ueda M (2012). Creation of pure nanodrugs and their anticancer properties. Angew Chem Int Ed.

[39] Zhou J, Li J, Du X, Xu B (2017). Supramolecular biofunctional materials. Biomaterials.

[40] Lee A (2021). Niraparib: a review in First-Line maintenance therapy in Advanced Ovarian Cancer. Target Oncol.

[41] Yang R, Wei T, Goldberg H, Wang W, Cullion K, Kohane DS (2017). Getting Drugs across Biological barriers. Adv Mater.

[42] Jain RK, Stylianopoulos T (2010). Delivering nanomedicine to solid tumors. Nat Reviews Clin Oncol.

[43] Gilbert DC, Chalmers AJ, El-Khamisy SF (2012). Topoisomerase I inhibition in Colorectal cancer: biomarkers and therapeutic targets. Br J Cancer.

[44] Rothkamm K, Barnard S, Moquet J, Ellender M, Rana Z, Burdak-Rothkamm S (2015). DNA damage foci: meaning and significance. Environ Mol Mutagen.

[45] Darden T, York D, Pedersen L (1993). Particle mesh Ewald: an N⋅log(N) method for Ewald sums in large systems. J Chem Phys.

[46] Ryckaert J-P, Ciccotti G, Berendsen HJ (1977). Numerical integration of the cartesian equations of motion of a system with constraints: molecular dynamics of n-alkanes. J Comput Phys.

