# Supplementary material for: A carrier-free supramolecular nano-twin-drug for overcoming irinotecan-resistance and enhancing efficacy against colorectal cancer
Source: J Nanobiotechnology. 2023 Oct 28;21:393. doi: 10.1186/s12951-023-02157-x (PMC10612220; doi:10.1186/s12951-023-02157-x)
Supplement: Supplementary file 1 — Supplementary Material 1 [file 12951_2023_2157_MOESM1_ESM.docx]

**Supporting Information**

A Carrier-Free Supramolecular Nano-Twin-Drug for Overcoming Irinotecan-Resistance and Enhancing Efficacy against Colorectal Cancer

Miaomiao Yuan ^1,3,#,^, Tong Chen ^3,#^, Lu Jin ^4,#^, Peng Zhang ^2,^*, Luoyijun Xie ^3^, Shuyi Zhou ^1^，Lianfeng Fan ^3^, Li Wang ^1^, Cai Zhang ^3^, Ning Tang ^1^, LiHao Guo ^1^, Chengmei Xie ^1^, Yanhong Duo ^5^, Ling Li ^3,^*, Leilei Shi ^1,3,^*

^1^ Precision Research Center for Refractory Diseases in Shanghai General Hospital, Shanghai Jiao Tong University School of Medicine, Shanghai 200025, China.

^2^ Department of Pharmacy, The Third Affiliated Hospital (The Affiliated Luohu Hospital) of Shenzhen University, 47 Youyi Road, Shenzhen 518001, China.

^3^ Department of pharmacology, the Eighth Affiliated Hospital, Sun Yat-sen University, Joint Laboratory of Guangdong-Hong Kong-Macao Universities for Nutritional Metabolism and Precise Prevention and Control of Major Chronic Diseases, Shenzhen, China.

^4^ School of Pharmaceutical Sciences, Sun Yat-sen University, Guangzhou 510006, China.

^5^Department of Microbiology, Tumor and Cell Biology (MTC), Karolinska Institutet, Stockholm, Sweden.

^#^ Miaomiao Yuan, Tong Chen and Lu Jin contributed equally to this work.

*Correspondence author.

Email: pzhang898018@163.com (Peng Zhang), lhlll0426@163.com (Ling Li), leilei.shi@shgh.cn (Leilei Shi)


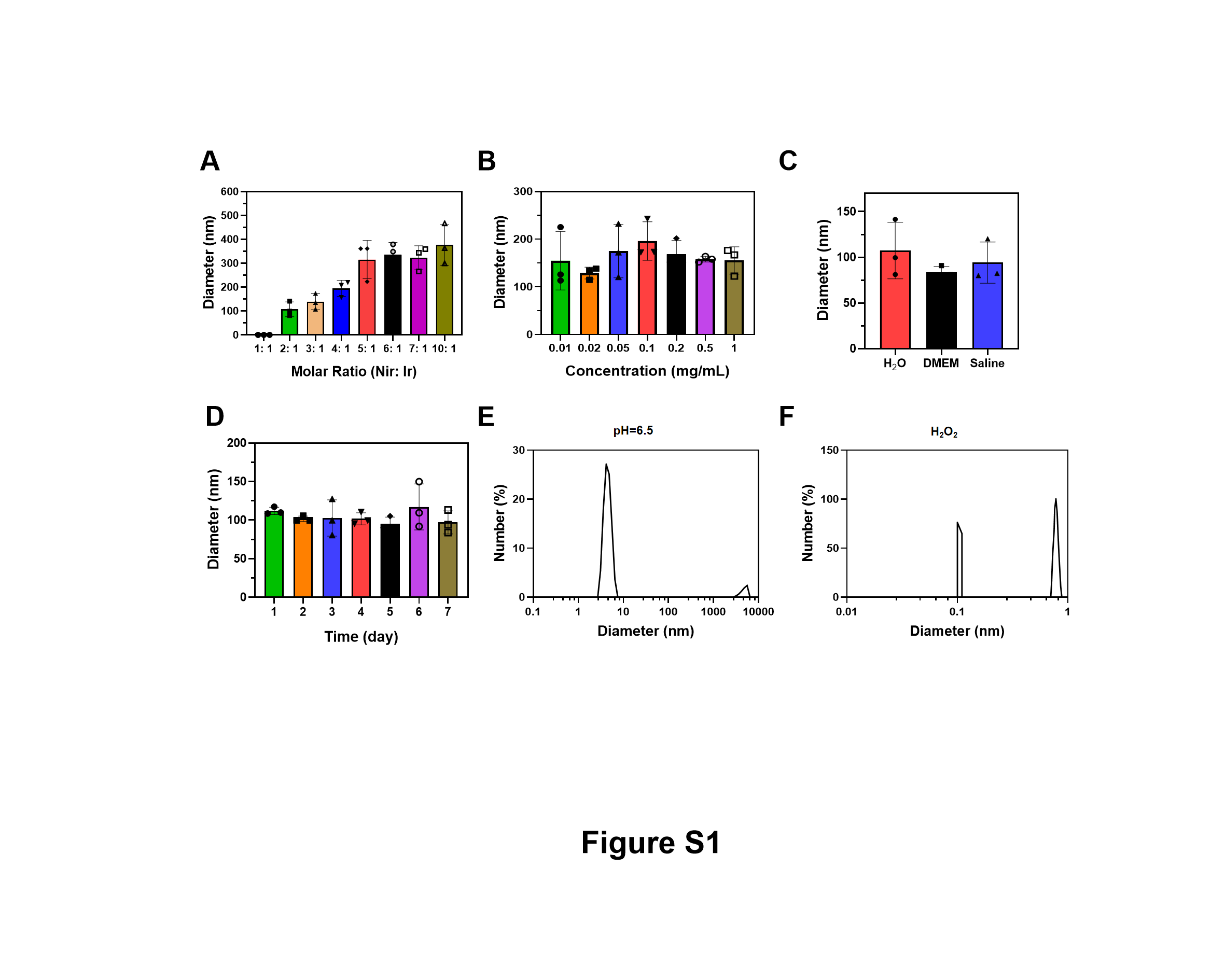


**Fig. S1.** Stability studies of Nir-Ir NPs. A) The hydrodynamic size of Nir-Ir NPs (1mg/mL in water) with different molar ratios of Ir to Nir determined by dynamic light scattering. Data were presented as mean values ± SD (n = 3 independent samples). B) The hydrodynamic size of Nir-Ir NPs with different concentrations in water determined by DLS. Data were presented as mean values ± SD (n = 3 independent samples). C) The hydrodynamic size of Nir-Ir NPs (1mg/mL) in different solutions. Data were presented as mean values ± SD (n = 3 independent samples). D) The hydrodynamic size of Nir-Ir NPs (1mg/mL) at different time points after preparation was determined. E) The hydrodynamic size of Nir-Ir NPs (1mg/mL) at pH 6.5. F) The hydrodynamic size of Nir-Ir NPs (1mg/mL) added with 10 μL H_2_O_2_ (30% v/v). Data were presented as mean values ± SD (n = 3 independent samples).


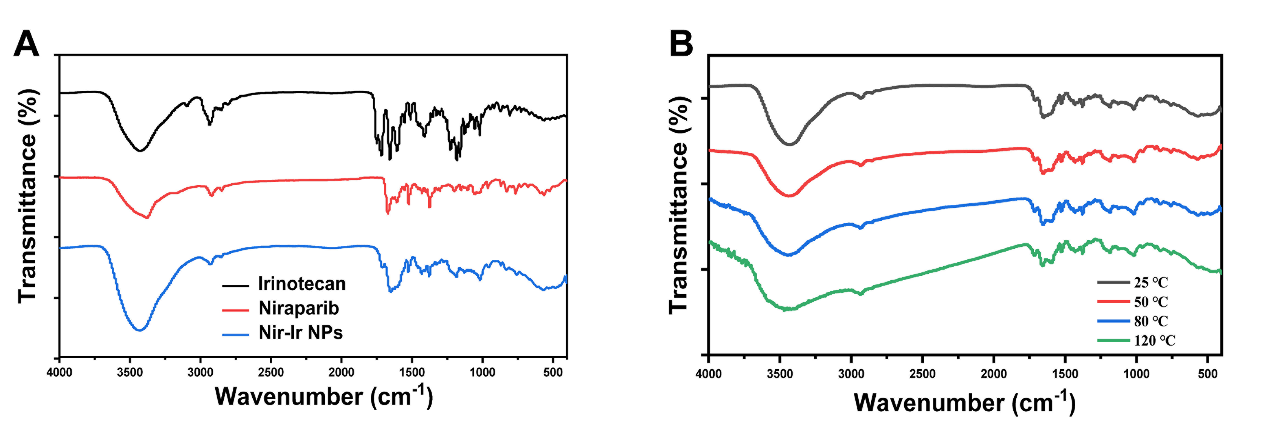


**Fig. S2.** Fourier transform infrared spectroscopy (FTIR) of Nir-Ir NPs. A) Representative infrared absorption spectrum of Ir, Nir and Nir-Ir NPs at room temperature. B) Representative variable temperature FTIR spectra of Nir-Ir NPs measured from 25 °C to 120 °C. Images were representative of three independent samples.

**
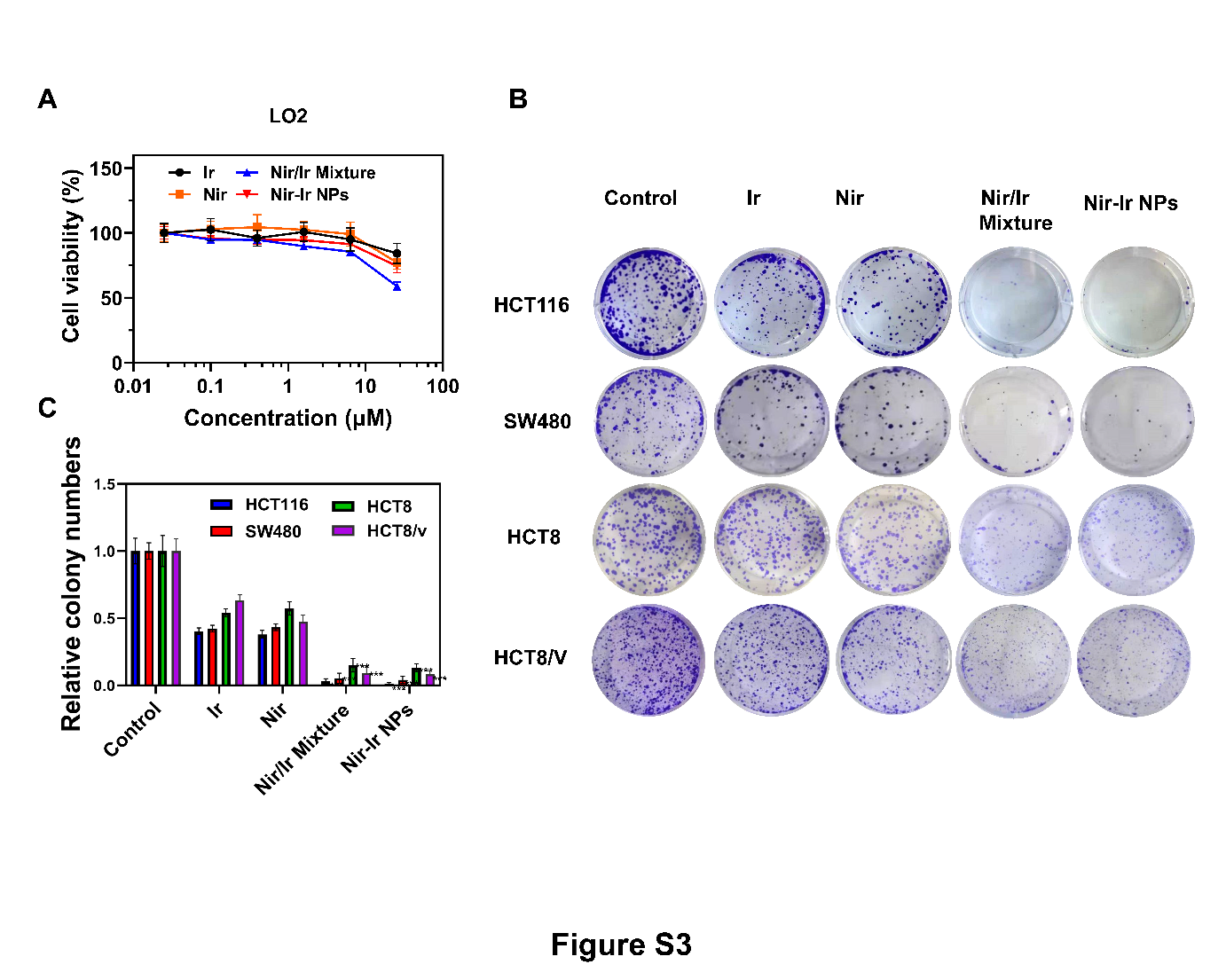
**

**Fig. S3.** A) Cell viability evaluation of Ir, Nir, Nir/Ir mixture and Nir-Ir NPs on LO2 cells. B) Colony assays of four CRC cell lines treated by Ir, Nir, Nir/Ir mixture and Nir-Ir NPs, respectively. Images were representative of three independent samples. C) Colony numbers (normalized on control) across the panel, with each bar corresponding to the mean of at least three experiments performed in duplicate. Data were presented as mean values ± SD (n = 3 independent samples). ****P* < 0.001, indicated statistical difference between groups from two-tailed student’s *t-test*.

**
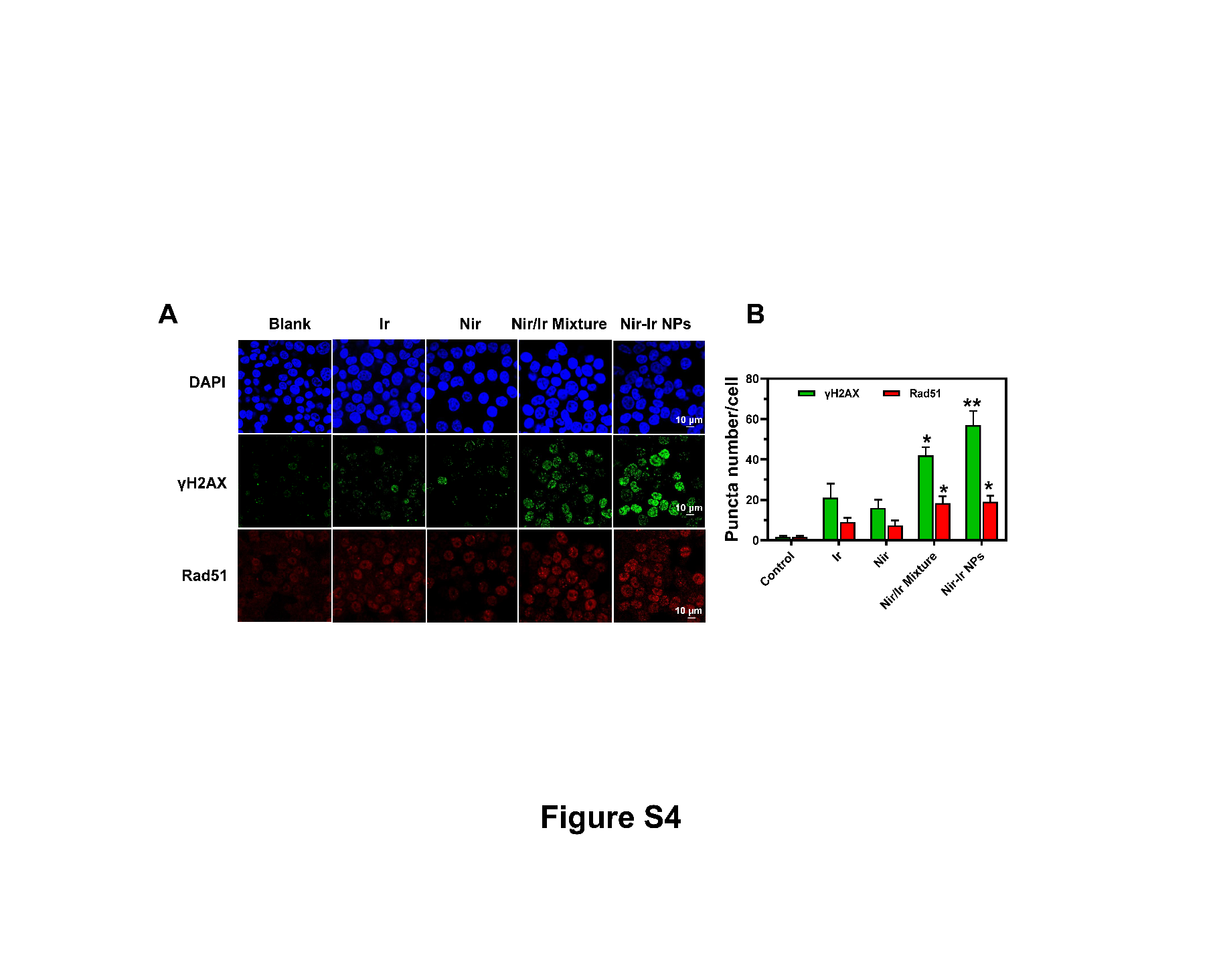
**

**Fig. S4.** Immunofluorescence and Immunoblot analysis of HCT116 cells treated with different drugs. A) HCT116 cells were immunostained for γH2AX and Rad51 after 24 h incubation with Ir, Nir, Nir/Ir mixture and Nir-Ir NPs. B) Numbers of γH2AX and Rad5 foci in HCT116 cells after various treatments for 24 h. Data were presented as mean values ± SD (n = 3 independent samples). **P* < 0.05, ***P* < 0.01 indicated statistical difference between groups from two-tailed student’s *t* test.

**
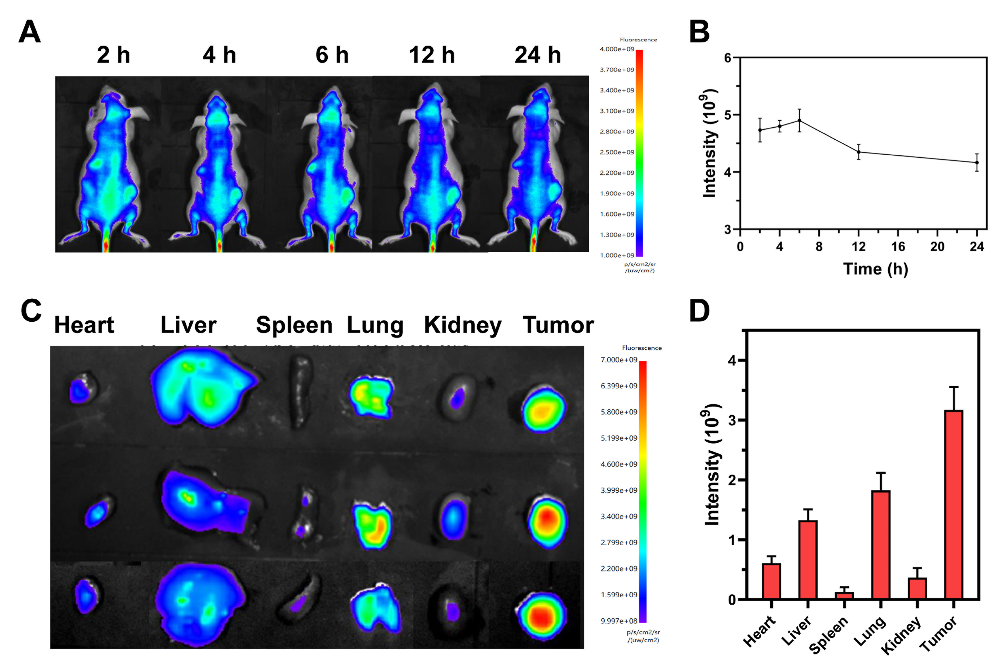
**

**Fig. S5.** *In vivo* distribution of Nir-Ir NPs in an HCT116 tumor xenograft-bearing mouse model. A) Representative *in vivo* fluorescence images of mice bearing subcutaneous HCT116 tumors after intravenous injection with Nir-Ir NPs/Cy5.5. Whole-body imaging was performed at predetermined times. B) Quantitative analysis of the fluorescence signals in the tumor site at different time points. Data were presented as mean values ± SD (n = 3 mice per group). C) Fluorescence images of *ex vivo* organs and tumors harvested at 24 h post-injection. D) Quantitative analysis of the fluorescence signals of *ex vivo* organs and tumors. Data were presented as mean values ± SD (n = 3 mice per group).


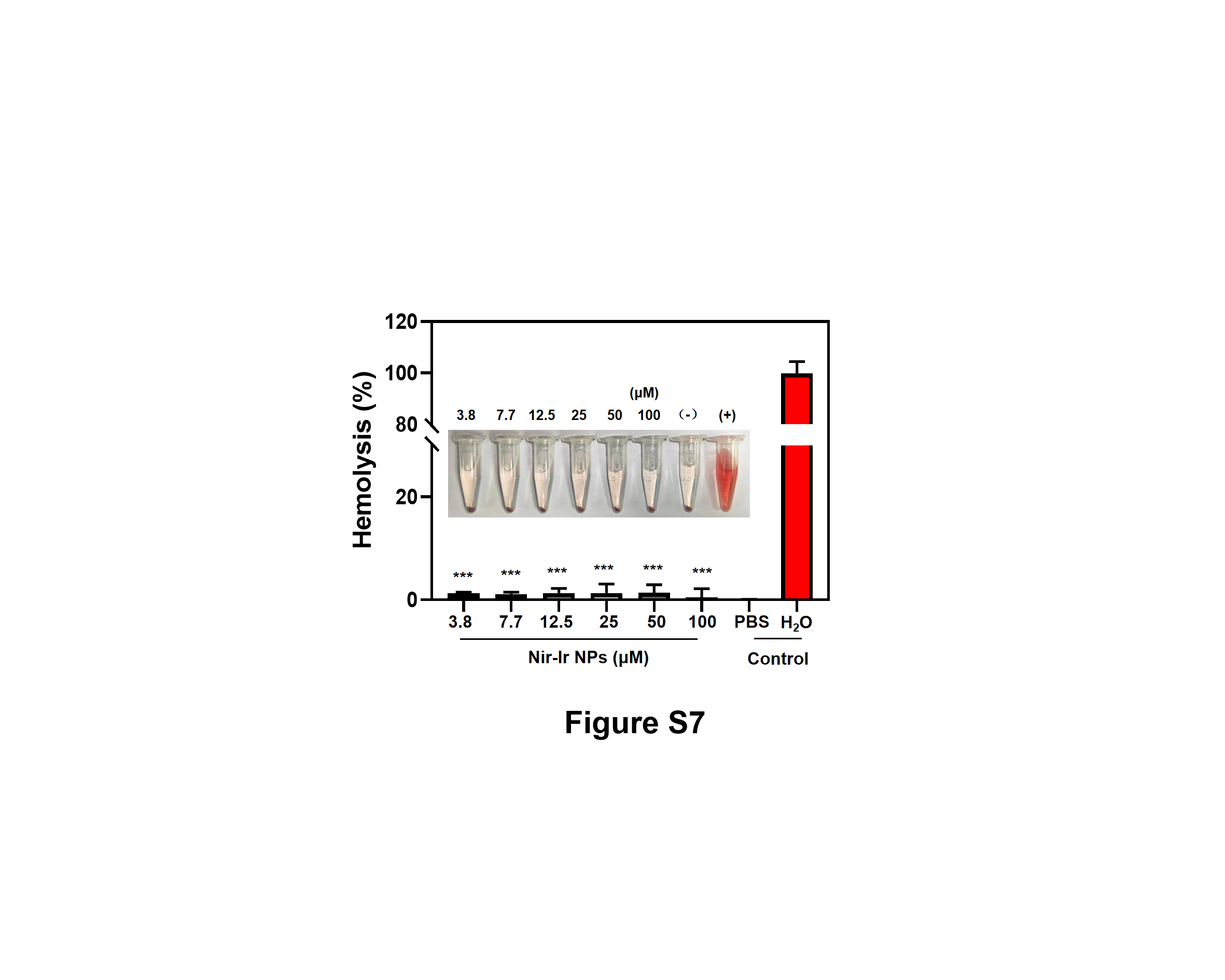


**Fig. S6.** *In vitro* hemocompatibility assay of Nir-Ir NPs at different concentrations (3.8, 7.7, 12.5, 25, 50, and 100μM). Co-incubation of PBS or H_2_O was set as negative control and positive control.

**
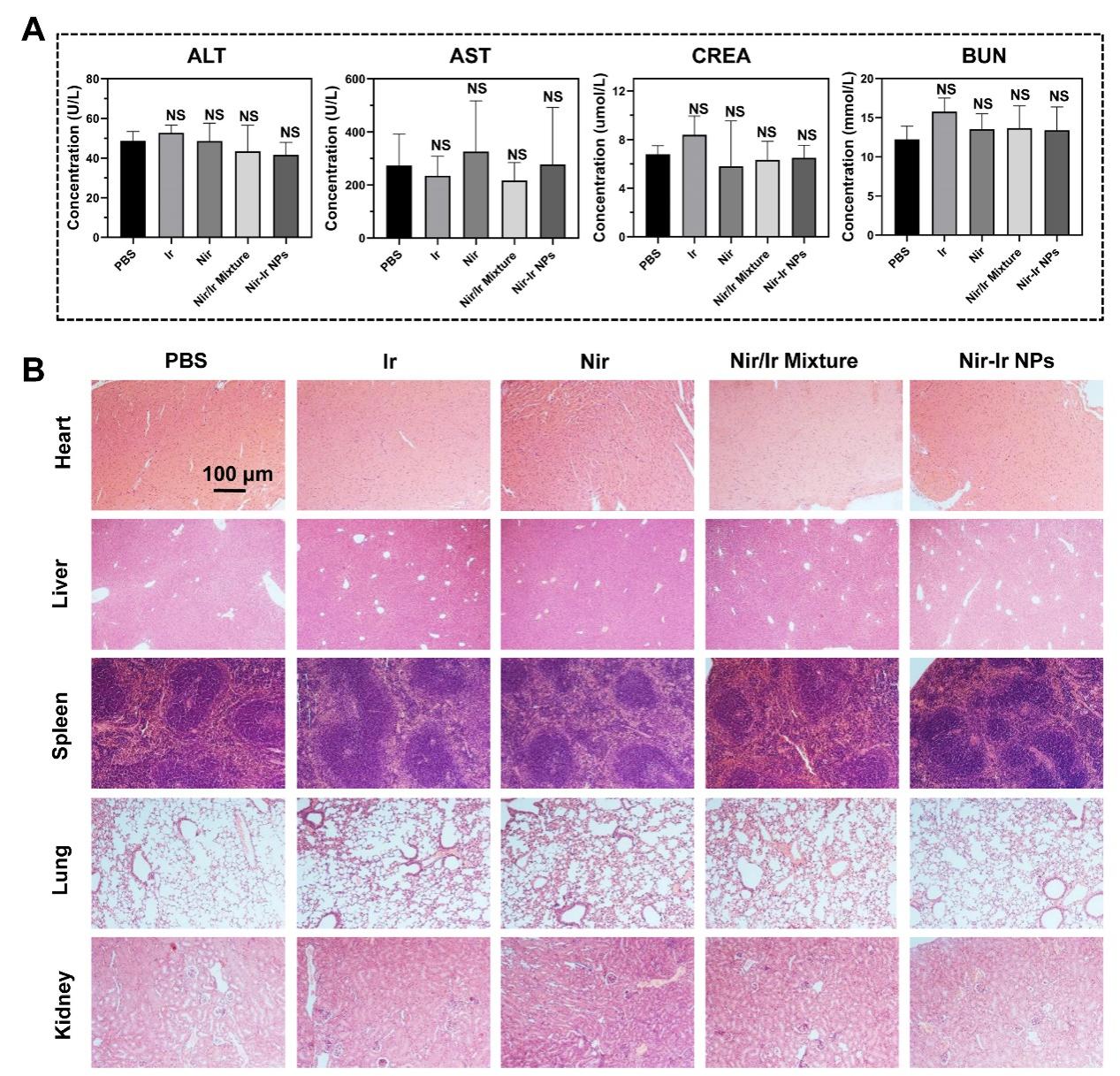
**

**Fig. S7.** *In vivo* biosafety study of different drugs in HCT116 subcutaneous tumor-bearing mice. A) Concentrations of ALT, AST, CREA and BUN in the serum of HCT116 subcutaneous tumor-bearing mice received different treatments. Data were presented as mean values ± SD (n = 4 independent samples). NS indicated non-significance. B) H&E images of the major organs of HCT116 tumor-bearing mice.

**Table S1.** IC50 values of the human CRC cell lines treated with free Ir, free Nir, Nir/Ir mixture and Nir-Ir NPs. The IC50 values were calculated from the *in vitro* cytotoxicity tests shown in Figure 3A-D.

|  | IC50 (μM) | | | |
| --- | --- | --- | --- | --- |
|  | Ir | Nir | Ir/Nir Mixture | Nir-Ir NPs |
| HCT116 | 1.58 | 4.12 | 0.44 | 0.41 |
| SW480 | 4.07 | 12.94 | 1.62 | 1.71 |
| HCT8 | 6.13 | 23.53 | 1.73 | 1.65 |
| HCT8/V | 21.35 | 22.78 | 4.07 | 4.15 |

.
